# A planar pentacoordinate oxygen in the experimentally observed [Be_5_O_6_]^2−^ dianion†

**DOI:** 10.1039/d5sc02361k

**Published:** 2025-05-16

**Authors:** Rui Sun, Yang Yang, Xin Wu, Hua-Jin Zhai, Caixia Yuan, Yan-Bo Wu

**Affiliations:** a Key Laboratory of Chemical Biology and Molecular Engineering, Ministry of Education, Key Laboratory of Materials for Energy Storage and Conversion of Shanxi Province, Institute of Molecular Science, Shanxi University 92 Wucheng Road Taiyuan Shanxi 030006 People's Republic of China wyb@sxu.edu.cn cxyuan@sxu.edu.cn; b Basic Sciences Department, Shanxi Agricultural University 1 South Mingxian Road, Taigu Shanxi 030801 People's Republic of China

## Abstract

Small multiply charged anions (SMCAs) are exceptionally challenging to generate in gas-phase experiments due to the spontaneous detachment of excess electrons. The [Be_5_O_6_]^2−^ dianion, first produced in 2006 *via* electrospray ionization and initially proposed by a concurrent computational study to adopt a linear O–Be-alternating structure, stands as a rare experimentally observed SMCA. In this study, by applying our recently developed electron-compensation strategy, we designed a starlike *D*_5h_ [O©Be_5_O_5_]^2−^ cluster featuring a planar pentacoordinate oxygen (ppO), which intriguingly shares the molecular formula [Be_5_O_6_]^2−^. Remarkably, this ppO isomer is not only 55.8 kcal mol^−1^ more stable than the previously reported linear isomer but also represents the global energy minimum on the [Be_5_O_6_]^2−^ potential energy surface. By adhering to the principles of the electron-compensation strategy, all Be atoms in the ppO isomer are electronically compensated and geometrically shielded by peripheral O atoms, resulting in a well-defined electronic structure. This is evidenced by a positive first vertical detachment energy of 2.44 eV, which effectively prevents the spontaneous loss of excess electrons. Thus, our work serendipitously uncovered and elaborately rationalized an experimentally unprecedented ppO within the previously generated SMCA [Be_5_O_6_]^2−^, marking a significant milestone in the field.

## Abstract 2

Sharing the molecular formula of [Be_5_O_6_]^2–^ generated by electrospray ionization in 2006, *D*_5h_ [O©Be_5_O_5_]^2–^ was identified here as a global energy minimum, representing the first experimental observation of a non-classical planar pentacoordinate oxygen.

## Graphical abstract

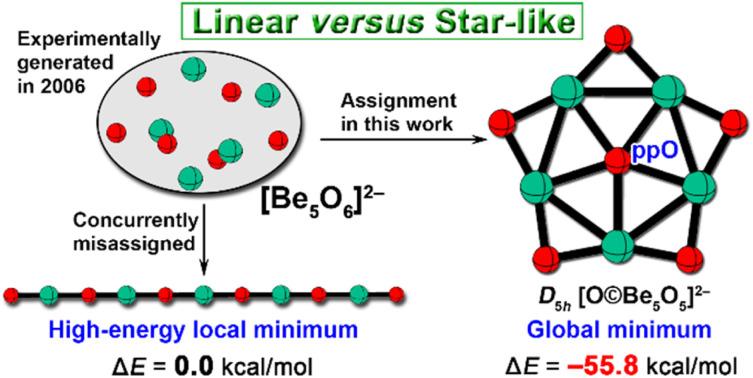

## Introduction

Clusters featuring non-classical bonding are typically generated and observed in the gas phase. In general, only the thermodynamically most stable isomer, known as the global energy minimum (GEM), survives the gas-phase annealing process and is subsequently detected through combined spectroscopy. In contrast, although significantly smaller amounts of local energy minima (LEMs) may persist, their spectroscopic signals are often overshadowed by those of the GEMs. For instance, nearly all clusters featuring non-classical planar hyper-coordination have been confirmed to be GEMs. Notable examples include [CB_4_]^+^,^1,2^ [CAl_4_]^−^,^3^ [CAl_4_Na]^−^,^4^ [CAl_3_X]^−/0^ (X = Si, Ge),^5,6^ [CAl_4_H]^−^,^7^ C_2_Al_4_,^8^ and [C_5_Al_5_]^−^ (ref. 9) with planar tetracoordinate carbon, [NAl_4_]^−/0^ (ref. 10 and 11) with a planar tetracoordinate nitrogen, [Al_4_X]^−/0^,^12^ [Al_3_X_2_]^−^ (X = Si, Ge),^13^ and [Cu_3_Si_3_]^−^ (ref. 14) with a planar tetracoordinate silicon or germanium, as well as [CoB_8_]^−/0^, [RuB_9_]^−^, [TaB_10_]^−^, and [NbB_10_]^−^ with planar octa-, nona-, and decacoordinate transition metals.^15,16^

Consequently, GEMs are highly favored in computational predictions of clusters with non-classical bonding due to their significantly greater compatibility with gas-phase experiments compared to LEMs. This preference is particularly evident in the design of clusters featuring non-classical planar hyper-coordination. Over the past two decades, hundreds of GEMs have been computationally predicted in this field,^17–23^ most of which exhibit planar pentacoordinate configurations involving H and typical second-row non-metals (ppX, where X = H, C, N, F, *etc.*).^23–35^ However, no cluster with a ppX has been experimentally observed to date. Furthermore, stable planar pentacoordinate oxygen (ppO) remains entirely unexplored, even among computationally predicted GEMs.

From the examples above, it is also evident that most experimentally observed clusters are monoanions, likely because they are characterized using photoelectron detachment spectroscopy, which favors monoanions. To the best of our knowledge, no small multiply charged anion (SMCA) with non-classical planar hyper-coordination has been reported, as SMCAs are exceptionally challenging to generate in gas-phase experiments due to the spontaneous detachment of excess electrons. Combined with the experimental gap concerning ppX, a significant breakthrough would be the observation of ppX in SMCAs.

**Fig. 1:**
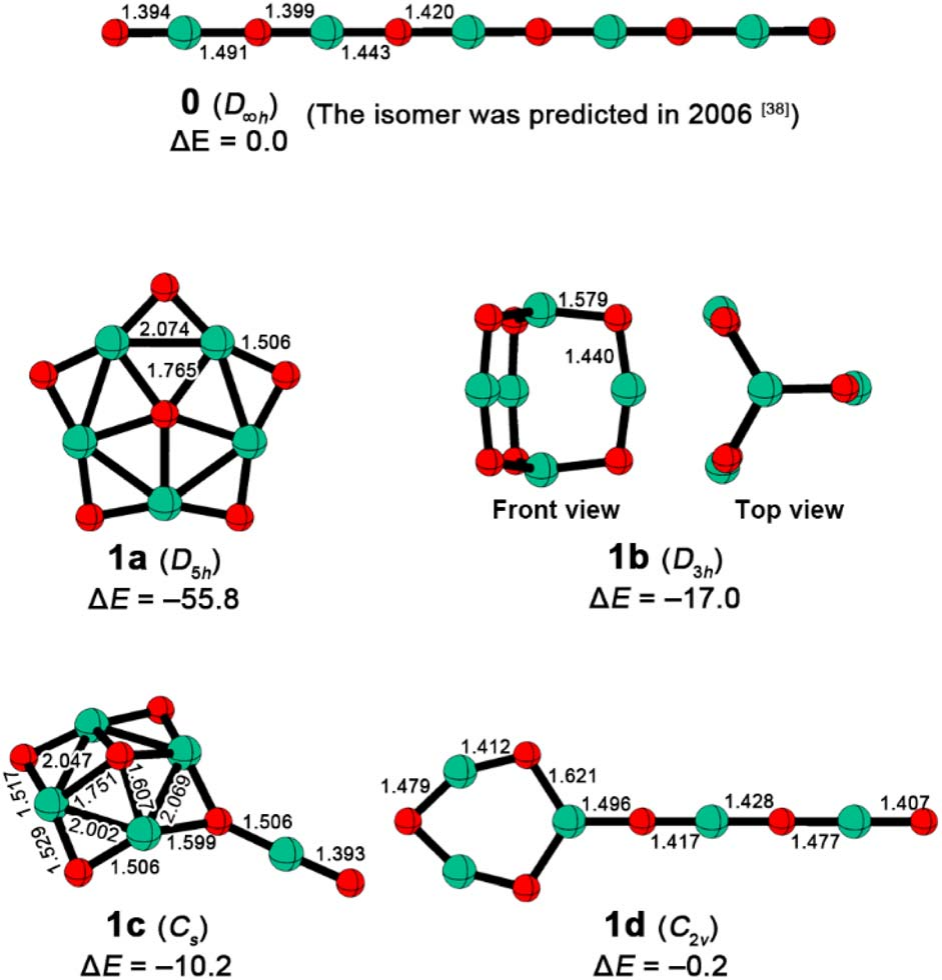
Optimized structures and relative energies (Δ*E*s) of low-lying isomers of Be_5_O_6_^2−^. The interatomic distances are given in Å, while the Δ*E*s are given in kcal mol^−1^ and computed using the energy of linear isomer 0 as the reference. Green and red balls denote Be and O atoms, respectively.

In this work, we report the design of a starlike *D*_5h_ [O©Be_5_O_5_]^2−^ cluster (1a in Fig. 1) featuring a planar pentacoordinate oxygen (ppO). By applying our recently developed electron-compensation strategy,^36^ this dianion exhibits a positive first vertical detachment energy of 2.44 eV, indicating the avoidance of spontaneous electron detachment. Notably, a literature survey revealed that the corresponding [Be_5_O_6_]^2−^ dianion was generated in 2006 *via* electrospray ionization,^37^ but a concurrent computational study^38^ incorrectly proposed a linear O–Be-alternating structure (0 in Fig. 1). Our ppO isomer is not only 55.8 kcal mol^−1^ more stable than such a linear isomer but also represents the GEM on the [Be_5_O_6_]^2−^ potential energy surface. Thus, we have identified a ppO within an experimentally observed SMCA, marking the first experimental observation of a cluster with a ppX and the first SMCA with planar hyper-coordination.

## Results and discussion

### Design of [O©Be_5_O_5_]^2−^

**Scheme 1:**
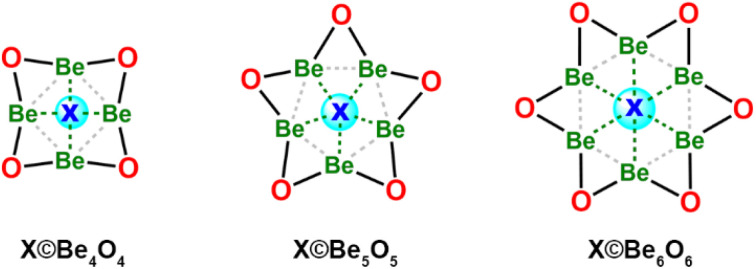
The X©Be_*n*_O_*n*_ (*n* = 4–6) model structures for designing the clusters with planar hypercoordinations.

The design of 1a originated from our systematic effort to create a beryllium-based starlike structure featuring a ppO, which coincidentally resulted in the molecular formula [Be_5_O_6_]^2−^. Specifically, we began by designing [X©Be_*n*_O_*n*_] model structures (see Scheme 1) using our recently proposed “electron-compensation” strategy. By following this approach, all Be atoms in the model structures are electronically compensated and geometrically shielded by peripheral O atoms, resulting in stable Be_*n*_O_*n*_ skeletons. Though these skeletons lack valence electrons for centripetal bonding, the Be atoms on such skeletons are ready to accept the donation from the central atom, that is, they are electronically suited to accommodate a central atom (X) with a fully filled valence shell, such as F^−^, O^2−^, N^3−^, Ne, and others. During the evaluation of the compatibility of these candidate atoms with Be_*n*_O_*n*_ skeletons for *n* = 4–6, we discovered that an oxygen atom could perfectly fit the Be_5_O_5_ skeleton at a molecular charge of −2.00 |*e*|, yielding a starlike dianion with *D*_5h_ symmetry, [O©Be_5_O_5_]^2−^ (1a). At the B2PLYP^39^-D3 (ref. 40) (BJ)^41^/aug-cc-pVTZ level of theory, 1a was confirmed as an energy minimum, with the lowest vibrational frequency (*ν*_min_) at 114 cm^−1^. The distances between the central O atom and each peripheral Be atom are uniformly 1.765 Å, only 0.030 Å longer than the sum (1.735 Å) of the average radii of Be (1.061 Å) and O (0.674 Å) atoms in tetrahedrally bonded crystals.^42^ Thus, the central O atom can be considered to be coordinated by five Be atoms, confirming the formation of a non-classical ppO.

### Stability consideration

Next, we compared the thermodynamic stability of 1a with the previously reported linear isomer 0. We reoptimized the structure of 0 at the B2PLYP-D3(BJ)/aug-cc-pVTZ level and refined the energies of 0 and 1a using CCSD(T)/aug-cc-pVTZ single-point calculations based on the optimized geometries [abbreviated as CCSD(T)//B2PLYP-D3(BJ)]. Remarkably, 1a is 55.8 kcal mol^−1^ lower in energy than 0, indicating that 1a should be significantly more likely to be generated and characterized in the gas phase experiment. To further assess the experimental viability of 1a, we extensively explored the singlet and triplet potential energy surfaces of [Be_5_O_6_]^2−^ using the stochastic search algorithm^43,44^ implemented in the GXYZ 3.0 program.^45^ The low-lying isomers are shown in Fig. 1. As illustrated, 1a was confirmed to be the exclusive GEM. In addition to 1a, we identified three new isomers (1b, 1c, and 1d) that are lower in energy than 0 by 17.0, 10.2, and 0.2 kcal mol^−1^, respectively. Structurally, 1b adopts a *D*_3h_ geometry with two axial beryllium atoms linked by three O–Be–O bridges, 1c features a *C*_s_ structure with a BeO group attached to a pyramidal Be_4_O_5_ moiety, and 1d consists of a Be_3_O_3_ ring with a linear Be_2_O_3_ tail. Note that the T1 diagnostic values of CCSD(T) calculations for these [Be_5_O_6_]^2−^ isomers range from 0.016 to 0.018, lower than the threshold of 0.020, so the results from such single-reference calculations should be reliable. Given that 1a is at least 38.8 kcal mol^−1^ lower in energy than its isomers, it should be the only experimentally observable isomer, dominating the [Be_5_O_6_]^2−^ dianion generated in electrospray ionization experiments. Therefore, we focus on 1a in the subsequent analysis.

**Fig. 2:**
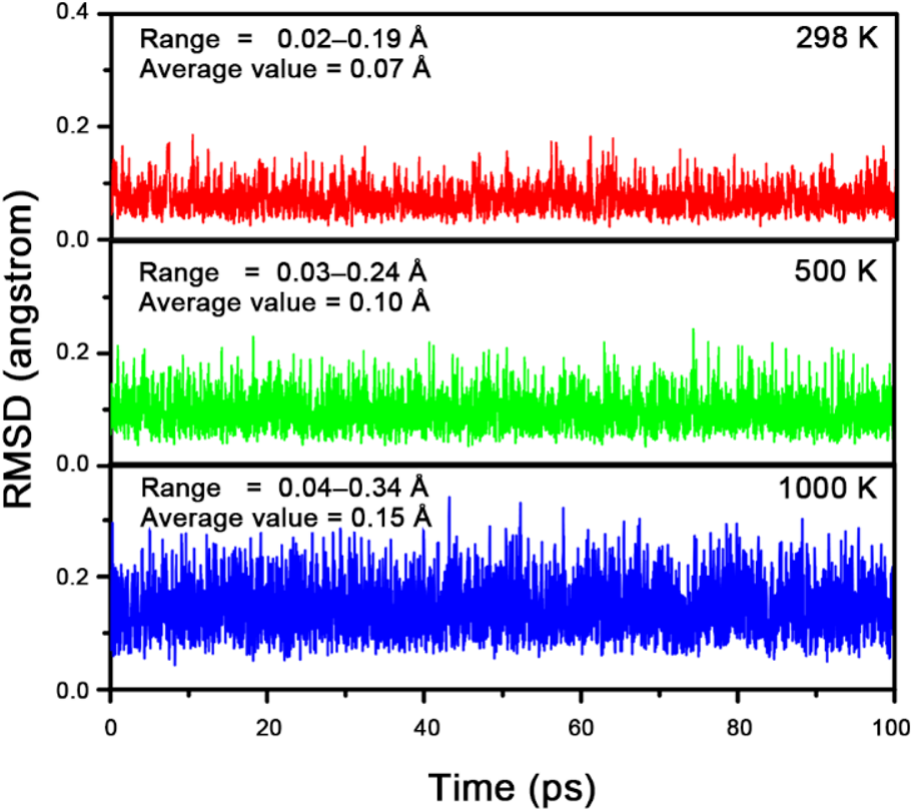
RMSD (in Å) *versus* simulation time (in ps) for the BOMD simulations (at 298, 500 and 1000 K) of 1a at the PBE/DZVP level.

To evaluate the dynamic stability of 1a,^46–49^ we performed Born–Oppenheimer molecular dynamics (BOMD) simulations. The structural evolution was monitored using the root-mean-square deviation (RMSD) of atomic positions. As shown in Fig. 2, the RMSD plots for simulations at 298 K, 500 K, and 1000 K exhibit no irreversible upward jumps, and the fluctuations remain small, with variation ranges of 0.02–0.19 Å, 0.03–0.24 Å, and 0.04–0.34 Å, respectively. The average RMSD values are 0.07 Å, 0.10 Å, and 0.15 Å, indicating that 1a is dynamically rigid against isomerization and dissociation at these temperatures.

### Electronic structure analyses

The electronic stability of 1a was assessed by examining its vertical detachment energy (VDE) using the outer valence Green's function (OVGF) method at the OVGF/aug-cc-pVTZ level.^50^ The positive first VDE of 2.44 eV indicates that electron detachment from 1a is endothermic, explaining its stable existence in electrospray ionization experiments. Additionally, we observed a large HOMO–LUMO gap of 4.76 eV at the B2PLYP-D3(BJ)/aug-cc-pVTZ level, suggesting that electron excitation from occupied to Rydberg orbitals is relatively difficult. Together, the positive VDE and large HOMO–LUMO gap confirm the electronic robustness of 1a.

**Fig. 3:**
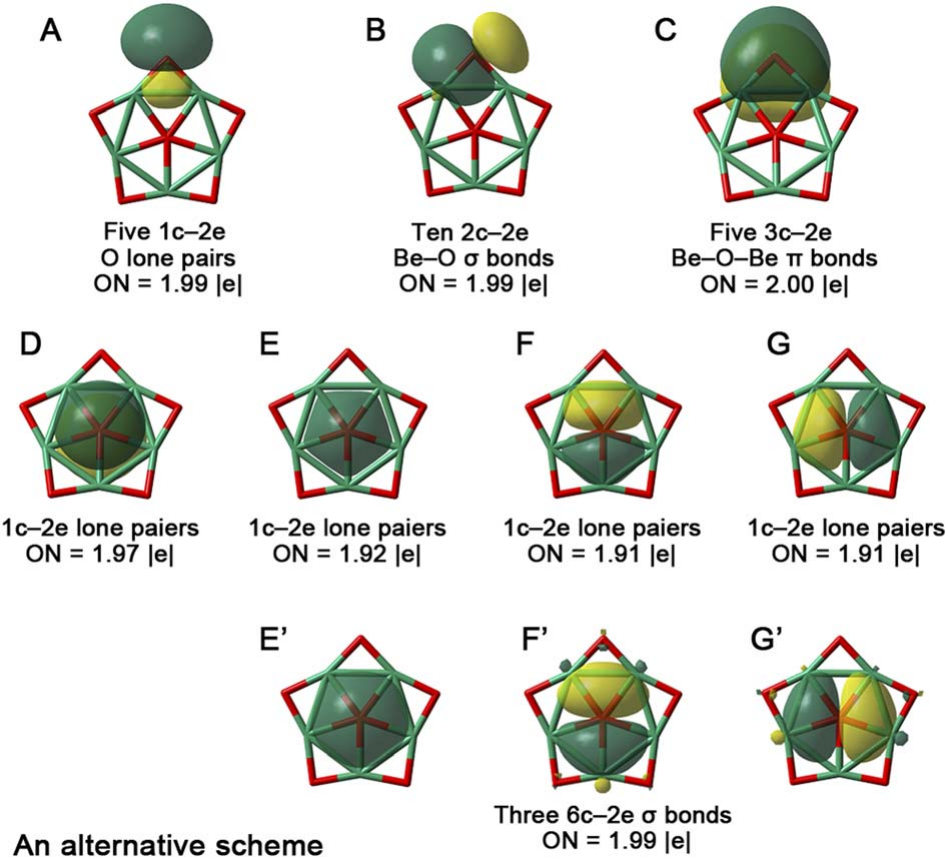
AdNDP view of chemical bonding in 1a. ON denotes the occupation number, which should be close to 2.00 |*e*| for doubly occupied orbitals.

To understand the stability of 1a, we analyzed its electronic structure using adaptive natural density partitioning (AdNDP)^51,52^ to identify characteristic *n*-center two-electron (*n*c-2e) bonds. As shown in Fig. 3, among the 24 valence electrons (including two negative charges) in 1a, there are five 1c–2e O lone pairs (occupation numbers, ONs = 1.99 |*e*|, orbital A), ten 2c–2e Be–O σ bonds (ONs = 1.99 |*e*|, orbital B), five 3c–2e Be–O–Be π bonds (ONs = 2.00 |*e*|, orbital C), and four lone pairs on the central ppO (ONs = 1.91–1.97 |*e*|, orbitals D–G). Notably, the formation of five 3c–2e Be–O–Be π bonds not only reinforces the Be_5_O_5_ skeleton but also compensates for the electron deficiency of the five Be atoms through O → Be π-backdonation, aligning with our electron-compensation strategy and stabilizing the starlike structure. Simultaneously, the central ppO satisfies the octet rule (with eight valence lone pair electrons). We also considered an alternative AdNDP scheme with three 6c–2e σ bonds (orbitals E′–G′). Although the ONs for E′–G′ (1.99 |*e*|) are slightly higher than those for E–G (1.91–1.92 |*e*|), the differences of 0.07–0.08 |*e*| are negligible, as they are distributed across five Be atoms (each contributing only 0.014–0.016 |*e*|). Thus, the AdNDP analysis confirms that the central ppO in 1a is a dianion.

To further confirm this, the natural bond orbital (NBO)^53^ analysis was performed for 1a at the B2PLYP-D3(BJ)/aug-cc-pVTZ level. Consistent with the AdNDP analysis, the ppO possesses the electron configuration of 2 s^1.91^2p_*x*_^1.94^p_*y*_^1.94^2p_*z*_^1.99^, suggesting a dianion. The natural charge on ppO (−1.80 |*e*|), while that on each Be atom is +1.71 |*e*|, suggesting strong electrostatic characteristics for ppO–Be interactions. Meanwhile, the Wiberg bond index for each ppO–Be bond is 0.08, indicating negligible covalent interactions.

To further investigate the nature of interactions between the central ppO and the peripheral Be_5_O_5_ skeleton, energy decomposition analysis with natural orbitals for chemical valence (EDA-NOCV)^54,55^ was performed at the B3LYP-D3(BJ)/TZ2P level. Table S1† presents the results of different fragmentation schemes. The scheme involving a singlet O^2−^ and a singlet neutral Be_5_O_5_ fragment yielded the lowest orbital interaction energy, consistent with the AdNDP analysis, and was therefore selected. As shown in Table 1, the total attractive energy (Δ*E*_attr_, −913.6 kcal mol^−1^) and the Pauli repulsion (Δ*E*_Pauli_, 475.3 kcal mol^−1^) result in an interaction energy (Δ*E*_int_) of −441.3 kcal mol^−1^. Within Δ*E*_attr_, electrostatic interactions (Δ*E*_elstat_, −656.7 kcal mol^−1^) account for 71.9%, while orbital interactions (Δ*E*_orb_, −256.9 kcal mol^−1^) contribute only 28.1%, indicating that the interaction between the central O^2−^ and the peripheral Be_5_O_5_ skeleton is predominantly electrostatic. Furthermore, 79.6% of the total Δ*E*_orb_ arises from terms exhibiting donation or backdonation characteristics (Δ*E*_orb(1)_–Δ*E*_orb(3)_ and Δ*E*_orb(5)_), whereas Δ*E*_orb(4)_, which reflects electron-sharing bond characteristics, contributes only 5.3% of Δ*E*_orb_. This further underscores the dianionic nature of the central ppO.

**Table 1:** Results of EDA-NOCV calculations for the Be_5_O_6_^2−^ cluster at the B3LYP-D3(BJ)/TZ2P level using O^2−^ (singlet, 2s^2^2p^6^) and the Be_5_O_5_ (singlet) ligand (L) as interacting fragments. Energies are given in kcal mol^−1^. The deformation densities of individual orbitals are given in the ESI^a^

Energy terms	Assignments	Interaction energies for O^2−^ + Be_5_O_5_
Δ*E*_int_		−441.3
Δ*E*_Pauli_		475.3
Δ*E*_attr_		−913.6
Δ*E*_elstat_		−656.7 (71.9%)^*a*^
Δ*E*_orb_		−256.9 (28.1%)^*a*^
Δ*E*_orb(1)_	L ← O (s) σ backdonation	−102.7 (40.0%)^*b*^
Δ*E*_orb(2)_	L ← O (p_*x*_) σ backdonation	−43.4 (16.9%)^*b*^
Δ*E*_orb(3)_	L ← O (p_*y*_) σ backdonation	−43.3 (16.9%)^*b*^
Δ*E*_orb(4)_	L–O (p_*z*_) electron-sharing bonds	−13.6 (5.3%)^*b*^
Δ*E*_orb(5)_	L → O (*p*_*x*_/*p*_*y*_) σ donation	−15.1 (5.8%)^*b*^
Δ*E*_rest_		−38.7 (15.1%)^*b*^

a *a* and *b* are the percentage contributions to Δ*E*_attr_ and Δ*E*_orb_, respectively.

## Conclusions

In summary, our targeted design of the starlike [O©Be_5_O_5_]^2−^ cluster, featuring a planar pentacoordinate oxygen (ppO), coincidentally shares the molecular formula [Be_5_O_6_]^2−^ with a small multiply charged anion generated in 2006 *via* electrospray ionization experiments. Through extensive exploration of the [Be_5_O_6_]^2−^ potential energy surface, we revealed that this ppO isomer is not only 55.8 kcal mol^−1^ more stable than the previously proposed linear O–Be-alternating isomer but also represents the global energy minimum. This strongly suggests that nearly all the experimentally generated [Be_5_O_6_]^2−^ clusters adopt the starlike ppO geometry. The experimental persistence of this dianion is attributed to the mitigation of electron deficiency of beryllium atoms through O → Be π-backdonation, as well as its unique electronic structure, which requires the two additional electrons (corresponding to the molecular charges) for the central ppO to satisfy the octet rule. Moreover, the VDE of 2.44 eV corresponds to an endothermic electron detachment for 1a. This energetically feasible transition implies that 1a is a promising candidate for observation *via* experimental photoelectron spectra. These findings not only uncover but also rationalize the experimental observation of an unprecedented ppO within a small multiply charged anion [Be_5_O_6_]^2−^, marking a significant breakthrough in the field.

## Computational

The singlet and triplet [Be_5_O_6_]^2−^ potential energy surfaces were explored using the stochastic search algorithm.^43,44^ Randomly generated structures were initially optimized at the B3LYP/6-31G(d) level and then the ten lowest-energy isomers were re-calculated at the B2PLYP^39^-D3 (ref. 40) (BJ)^41^/aug-cc-pVTZ level. The energies of the eight lowest isomers were further refined at the CCSD(T)/aug-cc-pVTZ level. The relative energies of the isomers were compared at the CCSD(T)/aug-cc-pVTZ level, incorporating B2PLYP-D3(BJ)/aug-cc-pVTZ zero-point energy corrections [abbreviated as CCSD(T)//B2PLYP-D3(BJ)]. The geometries of low-lying isomers are also re-optimized at the PBE0-D3/aug-cc-pVTZ level, which are essentially not different from those obtained at the B2PLYP-D3(BJ)/aug-cc-pVTZ level. The structures optimized at the B2PLYP-D3(BJ)/aug-cc-pVTZ level are shown in the text, while the optimized geometries (in Cartesian coordinates) are given in the ESI. Born–Oppenheimer molecular dynamic (BOMD) simulations^46–49^ were carried out to assess dynamic stability at the PBE/DZVP level. Vertical detachment energies (VDEs) were calculated using the outer valence Green's function (OVGF) procedure at the OVGF/aug-cc-pVTZ level.^50^ To better understand the chemical bonding, natural bond orbital (NBO)^53^ analysis and adaptive nature density partitioning (AdNDP) analysis^51^ were performed at the B2PLYP-D3(BJ)/aug-cc-pVTZ and B3LYP/6-31G* levels, respectively. The AdNDP analysis was done using the AdNDP program,^52^ while the energy decomposition analysis with natural orbitals for chemical valence (EDA-NOCV)^54,55^ was further conducted at the B3LYP-D3(BJ)/TZ2P level using the ADF 2022 program package.^56^ The stochastic search algorithm was implemented using the GXYZ 3.0 program,^45^ the CCSD(T) calculations were carried out using the MolPro 2012.1 package,^57^ and all other calculations were performed using the Gaussian 16 package.^58^

## Author contributions

The manuscript was written through contributions from all authors. All authors have given approval to the final version of the manuscript.

## Conflicts of interest

The authors declare no conflict of interest.

## Supplementary Material

SC-016-D5SC02361K-s001

## Data Availability

The data supporting this article have been included as part of the ESI.†
