# Supplementary material for: A planar pentacoordinate oxygen in the experimentally observed [Be5O6]2− dianion
Source: Chem Sci. 2025 May 16;16(28):12873–8. doi: 10.1039/d5sc02361k (PMC12091056; doi:10.1039/d5sc02361k)
Supplement: SC-016-D5SC02361K-s001 [file SC-016-D5SC02361K-s001.pdf]

Supplementary Information (SI) for Chemical Science.
This journal is © The Royal Society of Chemistry 2025

Electronic Supplementary Information

A planar pentacoordinate oxygen in the experimentally observed $[Be_5O_6]^{2-}$ dianion

Rui Sun, [a, b] Yang Yang, [a] Xin Wu [b] Hua-Jin Zhai, [a] Caixia Yuan *[a] and Yan-Bo Wu *[a]

[a] Key Laboratory of Chemical Biology and Molecular Engineering, Ministry of Education, Key Laboratory of Materials for Energy Storage and Conversion of Shanxi Province, Institute of Molecular Science, Shanxi University, 92 Wucheng Road, Taiyuan, Shanxi, 030006, P. R. China.

[b] Basic Sciences Department, Shanxi Agricultural University, 1 Mingxian South Road, Taigu, Shanxi, 030801, P. R. China.

*To whom correspondence should be addressed.

E-mail: cxyuan@sxu.edu.cn and wyb@sxu.edu.cn

Contents

Tbale S1: The EDA results of $[Be_5O_6]^{2-}$ cluster using different charged fragments at the B3LYP-D3(BJ)/TZ2P level. The most favourable fragments which are given by the smallest ΔE_{orb} value are shown *in* red. $\Delta E_{steric} = \Delta E_{pauli} + \Delta E_{elstat}$. Energy values are given in kcal/mol. (S: singlet; D: doublet; T: triplet)

Fig. S1: The shapes of deformation densities ($\Delta\rho$) for EDA-NOCV analysis of $[Be_5O_6]^{2-}$ cluster. The isovalues of the surfaces are 0.003 for $\Delta\rho_{orb(1)}$, 0.001 for $\Delta\rho_{orb(2)–(3)}$, 0.0003 for $\Delta\rho_{orb(4)}$, and 0.0005 for $\Delta\rho_{orb(5)}$. The direction of charge flow is from red to blue. Energy values are given in kcal/mol.

Cartesian Coordinates for the structures shown in **Fig. 1**.

Table S1: The EDA results of $[Be_5O_6]^{2-}$ cluster using different charged fragments at the B3LYP-D3(BJ)/TZ2P level. The most favourable fragments which are given by the smallest ΔE_{orb} value are shown in red. ΔE_{steric} = ΔE_{pauli} + ΔE_{elstat}. Energy values are given in kcal/mol. (S: singlet; D: doublet; T: triplet)

Energy	O^{2-} (S) + Be_5O_5 (S)	O^{2-} (T) + Be_5O_5 (T)	O^{-} (D) + $Be_5O_5^{-}$ (D)
ΔE_{int}	–441.27	–2209.80	–223.57
ΔE_{pauli}	475.19	163.94	410.53
ΔE_{elstat}	–656.69	–542.89	–330.34
ΔE_{orb}	–256.88	–1827.97	–300.88
**Energy**	**O (T) + $Be_5O_5^{2-}$ (T)**	**O^{3-} (S) + $Be_5O_5^{+}$ (S)**	**O^{3-} (T) + $Be_5O_5^{+}$ (T)**
ΔE_{int}	–344.15	–1465.85	–6109.33
ΔE_{pauli}	294.43	480.46	355.31
ΔE_{elstat}	–120.87	–1208.55	–1261.48
ΔE_{orb}	–514.84	–734.87	–5200.28

Fig. S1: The shapes of deformation densities ($\Delta\rho$) for EDA-NOCV analysis of $[Be_5O_6]^{2-}$ cluster. The isovalues of the surfaces are 0.003 for $\Delta\rho_{orb(1)}$, 0.001 for $\Delta\rho_{orb(2)-(3)}$, 0.0003 for $\Delta\rho_{orb(4)}$, and 0.0005 for $\Delta\rho_{orb(5)}$. The direction of charge flow is from red to blue. Energy values are given in kcal/mol.

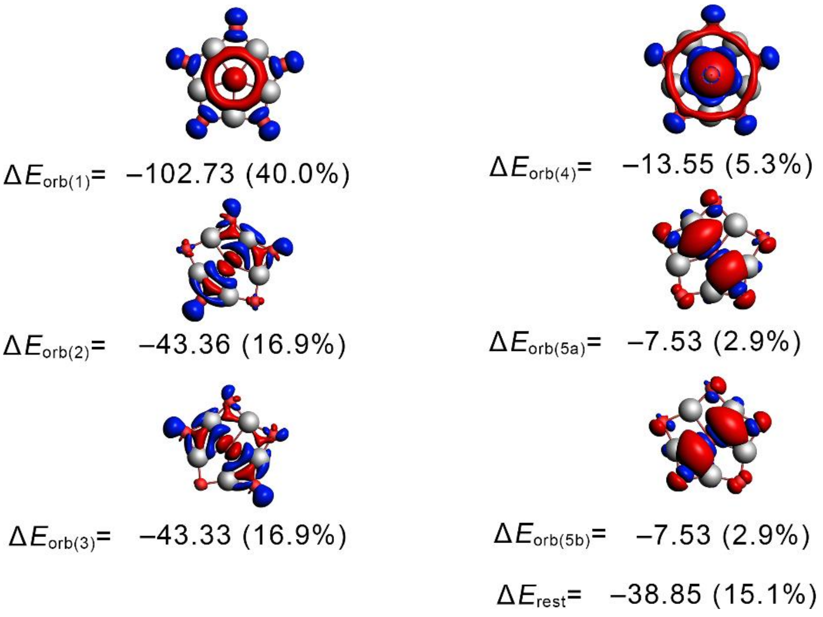

Cartesian Coordinates for the structures shown in **Fig. 1**.

B2PLYP-D3(BJ)/aug-cc-pVTZ-optimized structures (in Cartesian coordinates) shown in **Fig. 1**.

0

O	0.00000000	0.00000000	7.14759300
Be	0.00000000	0.00000000	2.86369100
Be	0.00000000	0.00000000	-5.75350500
Be	0.00000000	0.00000000	0.00000200
Be	0.00000000	0.00000000	-2.86369200
Be	0.00000000	0.00000000	5.75350400
O	0.00000000	0.00000000	-4.26291000
O	0.00000000	0.00000000	4.26290900
O	0.00000000	0.00000000	1.42029900
O	0.00000000	0.00000000	-7.14759400
O	0.00000000	0.00000000	-1.42029600

1a

O	0.00000000	0.00000000	0.00000000
Be	0.00000000	1.76451200	0.00000000
Be	-1.67815100	0.54526400	0.00000000
Be	-1.03715400	-1.42752000	0.00000000
Be	1.03715400	-1.42752000	0.00000000
Be	1.67815100	0.54526400	0.00000000
O	1.48087200	2.03824600	0.00000000
O	-1.48087200	2.03824600	0.00000000
O	-2.39610200	-0.77854100	0.00000000
O	0.00000000	-2.51941100	0.00000000
O	2.39610200	-0.77854100	0.00000000

1b

O	-1.35937000	-0.78483300	1.42614700
Be	0.00000000	1.77056600	0.00000000
Be	0.00000000	0.00000000	1.60003000
Be	-1.53335600	-0.88528300	0.00000000
Be	1.53335600	-0.88528300	0.00000000
Be	0.00000000	0.00000000	-1.60003000
O	-1.35937000	-0.78483300	-1.42614700
O	1.35937000	-0.78483300	-1.42614700
O	0.00000000	1.56966600	1.42614700
O	0.00000000	1.56966600	-1.42614700
O	1.35937000	-0.78483300	1.42614700

1c

O	-0.17525500	-1.31917600	0.00000000
Be	0.27882000	1.81402700	1.02352800
Be	0.27882000	1.81402700	-1.02352800
Be	0.27882000	-0.18770200	-1.03473300
Be	0.27882000	-0.18770200	1.03473300
Be	-0.61384700	-2.76018700	0.00000000
O	-0.94827900	-4.11287900	0.00000000
O	1.14295500	0.68704100	0.00000000
O	-0.01161700	2.89546800	0.00000000
O	-0.12926000	0.80165700	-2.09435300
O	-0.12926000	0.80165700	2.09435300

1d

O	0.00000000	1.36372800	-2.06613100
Be	0.00000000	0.00000000	-1.18999600
Be	0.00000000	-1.02468300	-3.43658900

Be	0.00000000	0.00000000	1.72314400
Be	0.00000000	0.00000000	4.62789800
Be	0.00000000	1.02468300	-3.43658900
O	0.00000000	0.00000000	-4.50292800
O	0.00000000	0.00000000	6.03463300
O	0.00000000	0.00000000	3.15088500
O	0.00000000	0.00000000	0.30573900
O	0.00000000	-1.36372800	-2.06613100

PBE0-D3/aug-cc-pVTZ-optimized structures (in Cartesian coordinates) shown in **Fig. 1**.

0

O	0.00000000	0.00000000	-7.11386400
Be	0.00000000	0.00000000	-2.85236000
Be	0.00000000	0.00000000	5.73032200
Be	0.00000000	0.00000000	0.00000000
Be	0.00000000	0.00000000	2.85236000
Be	0.00000000	0.00000000	-5.73032200
O	0.00000000	0.00000000	4.24609800
O	0.00000000	0.00000000	-4.24609800
O	0.00000000	0.00000000	-1.41485400
O	0.00000000	0.00000000	7.11386400
O	0.00000000	0.00000000	1.41485400

1a

O	-0.00001900	0.00000000	0.00000000
Be	-1.75112900	0.00000000	0.00000000
Be	-0.54112800	1.66542900	0.00000000
Be	1.41670000	1.02929200	0.00000000
Be	1.41670000	-1.02929200	0.00000000
Be	-0.54112800	-1.66542900	0.00000000
O	-2.02873900	-1.47397400	0.00000000
O	-2.02873900	1.47397400	0.00000000
O	0.77491600	2.38492500	0.00000000
O	2.50765800	0.00000000	0.00000000
O	0.77491600	-2.38492500	0.00000000

1b

O	-1.35454900	-0.78204900	1.42225700
Be	0.00004300	1.74911800	0.00000000

Be	0.00000000	0.00000000	1.58824700
Be	-1.51480200	-0.87452200	0.00000000
Be	1.51475900	-0.87459600	0.00000000
Be	0.00000000	0.00000000	-1.58824700
O	-1.35454900	-0.78204900	-1.42225700
O	1.35454900	-0.78204900	-1.42225700
O	0.00000000	1.56409900	1.42225700
O	0.00000000	1.56409900	-1.42225700
O	1.35454900	-0.78204900	1.42225700

1c

O	0.17396100	1.31296500	0.00000000
Be	-0.27366900	-1.80072400	1.01351100
Be	-0.27366900	-1.80072400	-1.01351100
Be	-0.27366900	0.18399500	-1.02785200
Be	-0.27366900	0.18399500	1.02785200
Be	0.60552800	2.75024000	0.00000000
O	0.93061300	4.09334500	0.00000000
O	-1.14114300	-0.68782600	0.00000000
O	0.01654000	-2.88255500	0.00000000
O	0.13230000	-0.79715900	-2.08428100
O	0.13230000	-0.79715900	2.08428100

1d

O	0.00000000	1.35857300	-2.05465900
Be	0.00000000	0.00000000	-1.18693200
Be	0.00000000	-1.01579300	-3.41724900
Be	0.00000000	0.00000000	1.71433600
Be	0.00000000	0.00000000	4.60741300
Be	0.00000000	1.01579300	-3.41724900
O	0.00000000	0.00000000	-4.48325000
O	0.00000000	0.00000000	6.00310800
O	0.00000000	0.00000000	3.13595200
O	0.00000000	0.00000000	0.30335100
O	0.00000000	-1.35857300	-2.05465900
